# Phylogeny, Systematics and Biogeography of the Genus *Panolis* (Lepidoptera: Noctuidae) Based on Morphological and Molecular Evidence

**DOI:** 10.1371/journal.pone.0090598

**Published:** 2014-03-06

**Authors:** Houshuai Wang, Xiaoling Fan, Mamoru Owada, Min Wang, Sören Nylin

**Affiliations:** 1 Department of Entomology, College of Natural Resources & Environment, South China Agricultural University, Guangzhou, Guangdong, China; 2 Department of Zoology, University of Stockholm, Stockholm, Sweden; 3 Department of Zoology, National Museum of Nature and Science, Tsukuba, Ibaraki, Japan; University of California, Berkeley, United States of America

## Abstract

The genus *Panolis* is a small group of noctuid moths with six recognized species distributed from Europe to East Asia, and best known for containing the widespread Palearctic pest species *P. flammea*, the pine beauty moth. However, a reliable classification and robust phylogenetic framework for this group of potentially economic importance are currently lacking. Here, we use morphological and molecular data (mitochondrial genes *cytochrome c oxidase subunit I* and *16S ribosomal RNA*, nuclear gene *elongation factor-1 alpha*) to reconstruct the phylogeny of this genus, with a comprehensive systematic revision of all recognized species and a new one, *P. ningshan* sp. nov. The analysis results of maximum parsimony, maximum likelihood and Bayesian inferring methods for the combined morphological and molecular data sets are highly congruent, resulting in a robust phylogeny and identification of two clear species groups, i.e., the *P. flammea* species group and the *P. exquisita* species group. We also estimate the divergence times of *Panolis* moths using two conventional mutation rates for the arthropod mitochondrial COI gene with a comparison of two molecular clock models, as well as reconstruct their ancestral areas. Our results suggest that 1) *Panolis* is a young clade, originating from the Oriental region in China in the Late Miocene (6–10Mya), with an ancestral species in the *P. flammea* group extending northward to the Palearctic region some 3–6 Mya; 2) there is a clear possibility for a representative of the Palearctic clade to become established as an invasive species in the Nearctic taiga.

## Introduction

The genus *Panolis* is a small group of noctuid moths, currently comprising six species which are known from Europe to East Asia and particularly concentrated in China [1]–[3]. Adults of this genus are medium-sized, often with sharply defined reniform stigma in the forewing. They usually emerge from February to May and are considered as characteristically early spring species.

The genus is best known for containing the Palearctic pest species *P. flammea*, the pine beauty moth, which has caused serious damage to pine forests [4], [5]. A reliable classification and phylogeny are indispensable for such a group of insects with economically important species. The genus was established by Hübner [6], and its type species *Noctua flammea* was explicitly designated by Hampson [7]. Subsequent studies on taxonomy of this genus were mainly focused on recognitions and descriptions of new taxa [1], [3], [8], [9] until Ronkay et al. [2] proposed a tentative classification framework based on morphological characters, and divided the genus into two species groups, the *P. flammea* species group and the *P. exquisita* species group. However, no study to date has been done to understand their phylogenetic relationships, and a comprehensive systematic review of this group is currently lacking.

The main objectives of this study are: 1) to provide a robust phylogeny of *Panolis* species for investigating their evolutionary relationships using morphological and molecular data; 2) provide a comprehensive systematic review of this genus with description of a new species; 3) to estimate the divergence times and reconstruct ancestral ranges of *Panolis* in order to infer their biogeographic history.

## Materials and Methods

### 1. Taxonomic study

All specimens examined were obtained from the Insect Collection, Department of Entomology, South China Agricultural University, Guangzhou, China (SCAU), and National Museum Nature and Science, Tsukuba, Japan (NSMT). The types of *P. exquisita* and *P. pinicortex* were examined in Zoological Research Museum Alexander Koenig, Bonn, Germany (ZFMK). The identification of two well-studied species, *P. flammea* and *P. japonica*, were based on Kononenko and Mikkola [1]. For *P. variegatoides* and *P. estheri*, clear illustrations of the holotypes can be found from Hacker [10] and Ronkay et al. [3], respectively. Types of the new species were deposited in SCAU and NSMT. Photos of adults were taken using a Nikon Coolpix S8000 digital camera after the wings were spread. Samples for studying wing venation and genitalia were prepared following Wang et al. [11], and were photographed using a Zeiss Discovery V12 stereoscope or a Leica EZ4 HD stereo microscope. Morphological terminology used in this study follows Ronkay et al. [2], except that the terminology of wing venation follows Holloway [12].

### 2. Nomenclature Acts

The electronic edition of this article conforms to the requirements of the amended International Code of Zoological Nomenclature, and hence the new names contained herein are available under that Code from the electronic edition of this article. This published work and the nomenclatural acts it contains have been registered in ZooBank, the online registration system for the ICZN. The ZooBank LSIDs (Life Science Identifiers) can be resolved and the associated information viewed through any standard web browser by appending the LSID to the prefix "http://zoobank.org/". The LSID for this publication is: urn:lsid:zoobank.org:pub:01DA739B-2961-4A9B-96CD-9FEA82E54217. The electronic edition of this work was published in a journal with an ISSN, and has been archived and is available from the following digital repositories: PubMed Central, LOCKSS.

### 3. Taxon sampling

All seven species of the genus *Panolis* were included for both morphological and molecular phylogenetic analyses, and the two Orthosiini species *Pseudopanolis heterogyna* and *Egira acronyctoides* were selected as outgroups. For morphological analysis, both male and female traits of all ingroup and outgroup species were sampled from our materials examined, except the female traits of *P. flammea* which were cited from Kononenko and Mikkola [1] and Ronkay et al. [2]. For molecular analysis, the taxa sampling is listed in Table S1.

### 4. Morphological data

A total of 18 adult morphological characters were evaluated and scored for the morphological phylogenetic analysis, 14 of which are binary and 4 are multistate. The data matrix is given in Table S2, and the characters and character states are in Appendix S1.

### 5. Molecular data

Total DNA was extracted from two or three legs of dried adult specimens using the TIANGEN DNA extraction kit following the manufacturer's instructions. The nucleotide sequences of two mitochondrial genes, *cytochrome c oxidase subunit I* (*COI*) and *16S ribosomal RNA* (*16S rRNA*), and one nuclear gene, *Elongation factor-1 alpha* (*EF-1a*), were selected for study. Primers used are shown in Table 1. Double-stranded DNA was amplified in 25 µ volume reactions containing 2.5 µ 10× buffer, 2 µ dNTP, 1 µ of each primer, 0.25 µ Taq DNA Polymerase (5 U/µl), 1 µ of extracted DNA, and ultrapure water. PCR cycle conditions for *COI* were an initial denaturation of 4 min at 94°C, 30 s at 94°C, 50 s at 47°C, and 1 min at 72 °C for 38 cycles, and a final extension at 72 °C for 5 min. The cycle conditions for *16S* were initial denaturation for 4 min at 94°C, 30 s at 94°C, 30 s at 47°C, and 1 min at 72 °C for 35 cycles, and a final extension for 5 min at 72 °C. Finally the cycle conditions for *EF-1a* were initial denaturation for 4 min at 94°C, 30 s at 94°C, 1 min at 55°C, and 2 min at 72°C for 35 cycles, and a final extension for 5 min at 72°C. The PCR products were purified with E.Z.N.A gel extraction kit (Omega), and were then sequenced on an ABI3730 sequencer directly or after cloned into pMD 18-T vector (TAKARA). All sequences were submitted to the GenBank database and their accession numbers are given in Table S1.

**Table 1:** Primers used in PCR and sequencing.

Gene	Primer name	Primer sequence (5′-3′)	Reference
*COI*	LCO1490	GGTCAACAAATCATAAAGATATTGG	Folmer et al.[13]
	HCO2198	TAAACTTCAGGGTGACCAAAAAATCA	Folmer et al.[13]
*16S rRNA*	LR-J-12887	CCGGTTTGAGCTCAGATCA	Simon et al. [14]
	LR-N-13398	CGCCTGTTTATCAAAAACAT	Simon et al. [14]
*EF-1a*	M44-1	CAGGAAACAGCTATGACCGCTGAGCGYGARCGTGGTATCAC	Cho et al. [15]
	rcM4	TGTAAAACGACGGCCAGTACAGCVACKGTYTGYCTCATRTC	Cho et al. [15]

### 6. Phylogenetic analyses

#### 6.1. Morphological data analysis

The morphological data was analyzed using Maximum parsimony in NONA v. 2.0 [16] as implemented in Winclada v.1.00.08 [17]. All characters were treated as non-additive. A initial heuristic search was executed with a Multiple TBR + TBR (multi*max*) search strategy and the default parameters, and then the Parsimony Ratchet analysis was done to collect all most parsimonious trees using 200 iterations, with one tree to hold per iteration, 10 characters to sample and 10 sequential ratchet runs. A strict consensus tree was generated from the Ratchet analysis. Bootstrap values were calculated using 1000 replications and a 10 search steps (mult*N) having one starting tree per replication, with random start and the option "Don't do max*(TBR)". Characters were optimized in Winclada v.1.00.08 and only the unambiguous characters were mapped on the strict consensus tree.

#### 6.2. Molecular data analyses

The sequences were aligned using Clustal W [18] as implemented in the program MEGA 4.0 [19], with default parameters. Gaps were treated as missing data in all analyses. The pairwise Kimura 2-Parameter (K2P) distances between species were calculated from the *COI* gene using MEGA 4.0. Phylogenetic congruence among *COI*, *16S rRNA*, and *EF-1a* genes was tested by the incongruence-length difference test (ILD) [20] in PAUP 4.0b10* [21].

Phylogenetic analyses were performed using the maximum parsimony (MP), maximum likelihood (ML) in PAUP 4.0b10*, and Bayesian inferring (BI) method in MrBayes 3.2.1 [22], [23]. For MP and ML analyses, the heuristic search was executed with 1000 random sequence additions and tree bisection reconnection (TBR) branch swapping. The optimal evolutionary model (GTR+I+G) for ML analysis was obtained using Akaike's information criterion (AIC) [24] implemented in jModelTest 0.1.1 [25]. Nodal support of MP and ML analyses was determined by 1000 bootstrap replicates. For BI analysis, the best-fit models of nucleotide substitution and model parameters for each gene region were selected with the jModelTest 0.1.1 under the AIC criterion. Two million generations were run along with two simultaneous iterations and four MCMC Markov chains. Trees were sampled every 1000 generations. The first 5000 sampled trees were discarded as burn-in, and the remaining trees were used to reconstruct a 50% majority-rule consensus tree and compute posterior probabilities. All trees were visualized with FigTree V. 1.4.0 (http://tree.bio.ed.ac.uk/software/figtree/).

#### 6.3. Combined data analyses

MP and BI analyses of the combined molecular and morphological dataset were performed in PAUP 4.0b10* and MrBayes 3.2.1, respectively. One individual for each species was used with all three gene regions (*COI*, *16S rRNA* and *EF-1a*) and an additional morphological partition included. For BI analysis, the ‘standard’ model implemented in MrBayes for analysis of discrete data was used for analysis of the morphological partition. The same MP and BI running parameters as mentioned in the section on molecular data analysis were employed.

### 7. Estimation of divergence times

Divergence times were estimated using only the 658bp *COI* gene in Beast V. 1.7.5 [26]. The HKY+G model was implemented, and the three codon positions were unlinked in order to be estimated independently. As there are no fossil records of reasonably closely related species that can be used to calibrate the tree topologies for this study, we used two conventional mutation rates for the arthropod mitochondrial *COI* gene from the published literatures: 2.3% (originally found by Brower [27] with similar rates later independently found in other insects, see below) and 1.5% [28], [29] per million years (per My). BEAST analyses were carried out under either a strict or lognormal relaxed clock to ascertain the effect of these two clock models on the resulting estimates of divergence times. Under the strict clock model, we fixed a rate of 0.0115 or 0.0075 substitutions/site/million years (corresponding to the divergence rate of 2.3% or 1.5% per million years, respectively). For the lognormal relaxed clock model, we assumed a normal distribution with a mean of 0.0115 or 0.0075, and set a standard deviation of 10%. A Yule speciation process model was selected for the tree prior, with a 50% majority-rule consensus tree obtained from Bayesian analysis of the combined molecular and morphological dataset as the input topology. Two independent MCMC analyses were run for 10,000,000 generations, with Markov chains sampled every 1,000 generations. The analysis results of the two runs were combined in LogCombiner v1.7.5 with the initial 10% of the trees discarded as burn-in. Tracer V. 1.5 [30] was used to determine convergence, measure the effective sample size of each parameter, and calculate the mean and 95% highest posterior density (HPD) intervals for divergence times. The consensus tree was compiled with TreeAnnotator 1.7.5 and displayed in FigTree V. 1.4.0. In addition, Bayes factors [31] were calculated using log marginal likelihood with stepping stone sampling for a comparison of the best fit model between the strict and lognormal relaxed clock.

### 8. Biogeographic analysis

**Figure 1:**
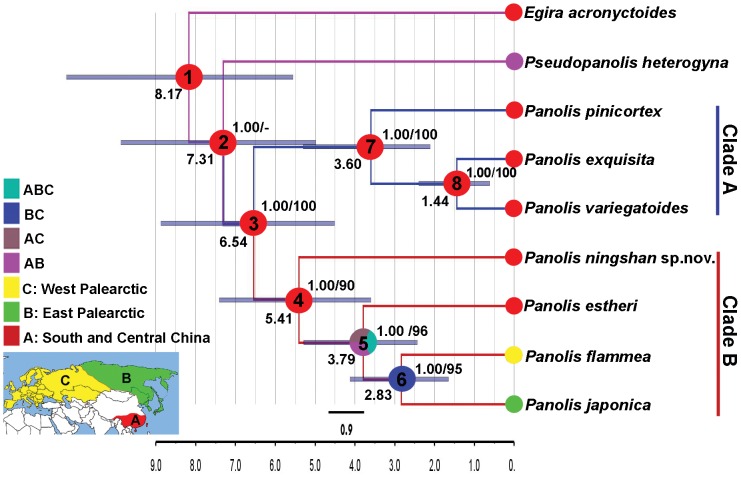
Phylogenetic tree obtained using the combined molecular and morphological data. The tree shown is 50% majority-rule consensus tree inferred from Bayesian analysis of the combined molecular and morphological data. Numbers at the right sides of the nodes indicate posterior probabilities and bootstrap values (PP/BP). BP values are shown when ≥50%. Numbers at the left sides of the nodes are estimates of mean divergence times (My) from the BEAST using the *COI* sequence only and calibrating with a rate of 0.0115 substitutions/site/My under the strict clock, with 95% HPD intervals indicated as blue bars. The small pie charts at terminal represent the distribution areas of each species. The larger pie charts at internal nodes represent the probabilities of ancestor area reconstructed from DIVA analysis. Node numbers are given in the larger pie charts.

In order to infer the biogeographic history of *Panolis* moths, we reconstructed ancestral areas using dispersal-vicariance analysis as implemented in the program DIVA 1.2 [32], [33], which is one of the most widely-used methods for the reconstruction of ancestral distribution in biogeographic studies [34]–[36]. The consensus tree generated from Bayesian analyses of the combined molecular and morphological dataset was selected for the optimal reconstruction of ancestral distribution. Analyses were performed using the default settings, with data matrices consisting of three geographic areas. These areas are defined based on the distribution information of the species (Fig. 1): A-South and Central China, with Qinling Mountain as boundary; B-East Palearctic, including northern east China, Korea, Japan and eastern Siberia; C-West Palearctic, ranging from Europe and western Siberia, with Yenisei River as a boundary.

## Results

### 1. Morphological data analysis

**Figure 2:**
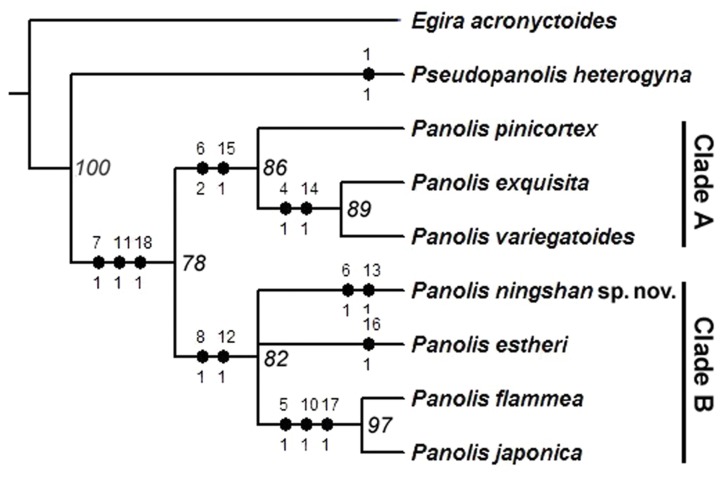
Phylogenetic tree obtained using morphological data. The strict consensus tree obtained from three equally most parsimonious trees based on the Parsimony Ratchet analysis (L = 23, CI = 95, RI = 95). Black circles represent nonhomoplasious changes (synapomorphies or autapomorphies). Numbers above the branches indicate character numbers, below the branches indicate character states. Bootstrap support values are shown on the right nodes with Italic numbers.

The parsimony analysis yielded three equally most parsimonious trees (tree length = 23, consistency index (CI = 95 and retention index (RI) = 95). The strict consensus tree is depicted in Fig. 2, which has one node collapsed, with the same values of tree length, CI and RI. In the tree obtained, the seven *Panolis* species form a monophyletic group, which is well supported by three synapomorphic characters: hindwing venation with Rs and M1 short stalked (7∶1), Ampulla short and round apically in the male genitalia (11∶1) and corpus bursae with signum in the female genitalia (18∶1). The group is divided into two clades. Clade A comprises *P. exquisita*, *P. variegatoides* and *P. pinicortex*. The monophyly of this clade is supported by light hindwing color (6∶2) and the posterior part of ductus brusae being short sclerotized (15∶1). Within clade A, *P. exquisita* is the sister species of *P. variegatoides*, which is supported by forewing with black antemedial and medial lines (4∶1) and carina with strongly sclerotized thorns (14∶1). The other four *Panolis* species form the clade B, which is supported by the uncus being narrow basally and slightly dilated apically (8∶1) and sacculus short (12∶1). Within the clade, *P.flammea* and *P. japonica* form a sister group based on forewing venation with R3 and R4 stalked about 1/2 (5∶1), harpe absent (10∶1) and posterior part of corpus bursae with sclerotized ribs (17∶1). However, the relationships between this sister group and the remaining two species are unresolved.

### 2. Molecular data analyses

The uncorrected pairwise genetic distances for 658 bp of the *COI* gene are given in Table S3. The interspecific genetic distances between ingroup taxa ranged from 1.9% (*P. exquisita* to *P. variegatoides*) to 8.2% (*P. japonica* to *P. pinicortex*). The two intraspecific sequences of *P. flammea* are identical, and so are those of the outgroup species *P. heterogyna.*

The concatenated dataset of three genes consisted of 2189 nucleotide positions (658 for *COI*, 465 for *16S rRNA* and 1066 for *EF-1a*, respectively), of which 261 are variable and 146 are parsimony-informative. The ILD test was not significant (P = 0.921), thus the three genes were combined into a single matrix for all subsequent phylogenetic analyses. Best-fit models and corresponding model parameters as estimated using jModeltest are provided in Table S4.

**Figure 3:**
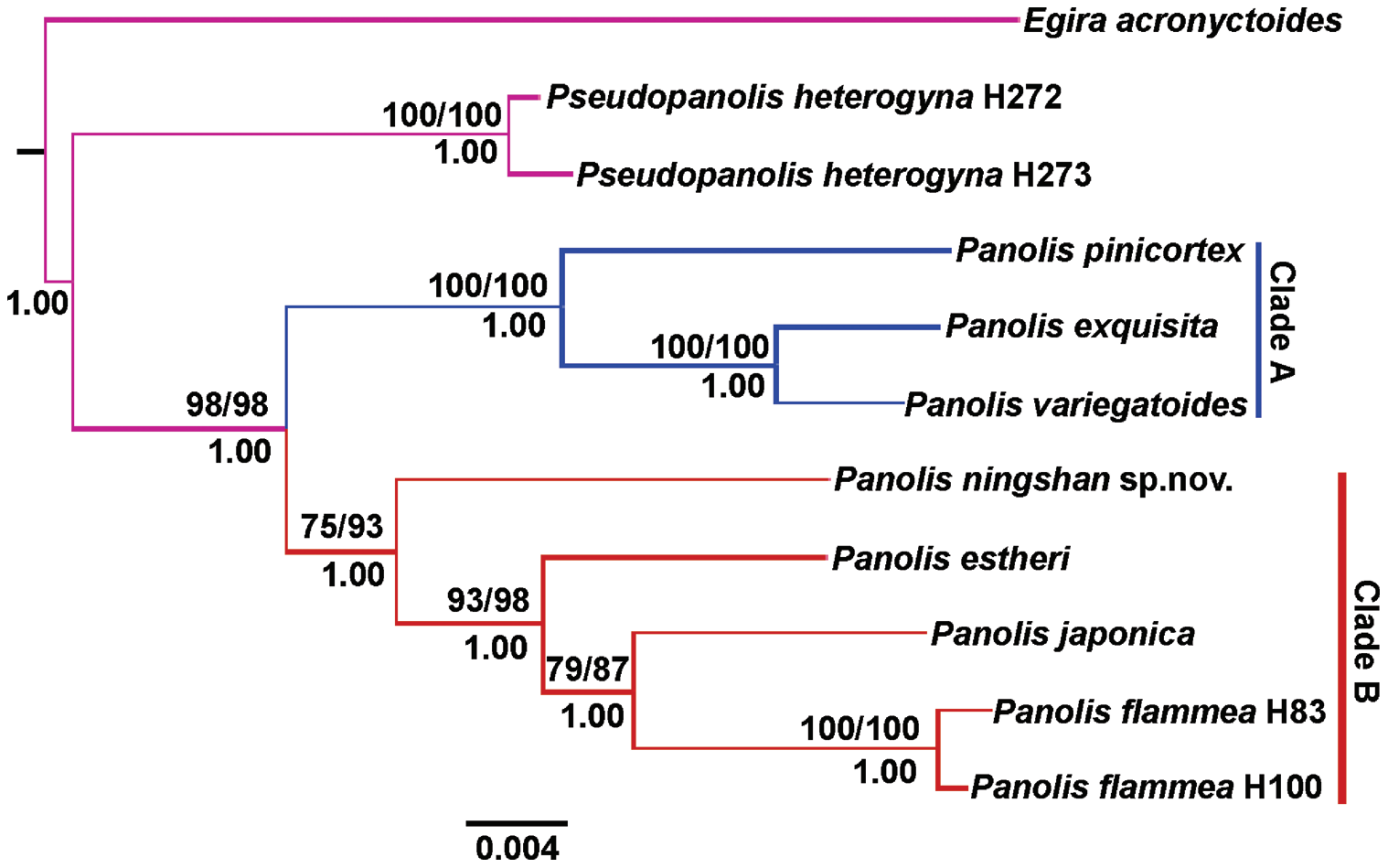
Phylogenetic tree obtained using molecular data. The 50% majority-rule consensus tree inferred from Bayesian analysis of the combined *COI*, *16S rRNA* and *EF-1a* data. Numbers above the branches indicate MP bootstrap values in ML and MP (MP/ML), bootstrap values are only shown when ≥50%. Numbers below the branches indicate posterior probabilities.

The maximum parsimony, Maximum likelihood and Bayesian analyses for *COI*+*16S*+*EF-1a* dataset resulted in similar tree topologies with only subtle differences in bootstrap and posterior probability support values (Fig. 3). The tree topologies show strong support for monophyly of the *Panolis* group (MP bootstrap value/ML bootstrap value/BI posterior probability = 98/98/1.00, respectively). Within the group, two well-supported clades are identified. Clade A comprises three species, *P. pinicortex* as sister to *P. exquisita*+ *P.variegatoides* and is strongly supported (100/100/1.00). Clade B consists of four species, with *P. ningshan* **sp. nov.** sister to *P. estheri*+*P. japonica*+*P. flammea*(75/93/1.00). *P. japonica* and *P. flammea* form a sister group (79/87/1.00).

### 3. Combined data analyses

The parsimony analysis of the combined morphological and molecular dataset generated a single tree with a length of 399, a CI of 0.797 and a RI of 0.625. Bayesian analysis yielded the same overall topology as the parsimony analysis, with higher posterior probabilities than bootstrap values in the clade B (Fig. 1). Both analysis results strongly support the phylogenetic relationships among *Panolis* species elucidated in the separate analysis of molecular data, and also well solve the relationships in clade B which were unresolved in the analysis of only morphological data.

### 4. Divergence time analyses

All BEAST analyses show high convergence, with effective sample size (ESS) values well above 189 for all parameters. The results for all age estimates are presented in Table 2, including 95% highest posterior density (HPD) intervals. A Bayesian topology is shown in Fig. 1, with the divergence times using the rate of 2.3% per million years under the strict clock.

**Table 2:** Estimates of divergence times for different nodes from BEAST using strict and relaxed clock at 2.3% and 1.5% per My.

Node number	Strict Clock (2.3% per My)	Lognormal relaxed clock (2.3% per My)	Strict Clock (1.5% per My)	Lognormal relaxed clock (1.5% per My)
	Mean (95% HPD)	Mean (95% HPD)	Mean (95% HPD)	Mean (95% HPD)
1	8.17 (-)	8.79 (-)	12.49 (-)	13.59 (-)
2	7.31 (4.99–9.87)	7.68(4.69–11.35)	11.18 (7.68–15.06)	11.87(7.09–17.65)
3	6.54 (4.52–8.87)	6.67(4.13–9.80)	10.00 (7.01–13.58)	10.32 (6.30–15.17)
4	5.41 (3.60–7.40)	5.48(3.22–8.10)	8.27 (5.61–11.31)	8.47 (4.93–12.47)
5	3.79 (2.43–5.29)	3.80(2.15–5.76)	5.81 (3.84–8.12)	5.89 (3.25–8.86)
6	2.83 (1.65–4.12)	2.80(1.38–4.44)	4.34 (2.54–6.26)	4.33 (2.09–6.80)
7	3.60 (2.11–5.30)	3.53(1.77–5.46)	5.51 (3.22–8.03)	5.46 (2.84–8.67)
8	1.44 (0.61–2.39)	1.54(0.50–2.75)	2.22 (0.93–3.68)	2.38 (0.83–4.35)

*Note: node numbers are shown in Fig. 1.

Times estimated using the strict and lognormal relaxed clocks at the same mutation rate (2.3% or 1.5 per My) are nearly concordant for *Panolis* group, with more wide 95% HPD intervals under the lognormal relaxed clock (Table 2). Bayes factors tests show that the strict clock is favored compared to the lognormal relaxed clock (2.3%, 2lnBF_A–B_ = 3.9201; 1.5%, 2lnBF_A–B_ = 5.0199).

The estimates of mean divergence time under the strict clock indicate that the first diversification (crown group age) of the genus *Panolis* was by the Late Miocene (6.54–10.00 Mya). The initial split of the Clade A occurred at estimated times ranging from 5.41 to 8.27 Mya, and later *P. estheri* diverged from *P. flammea* + *P. japonica* group at estimated times extending from 3.79 to 5.81 Mya. The latest split within the Clade A happened during the Pliocene (2.83–4.34 Mya). The Clade B first diversified at estimated times ranging from 3.60 to 5.51 Mya, which suggests a younger crown group age than for the Clade A. The sister group *P. exquisita* + *P. variegatoides* diverged in the Pleistocene, (1.44–2.22 Mya), and it is thus the youngest group in *Panolis*.

### 5. Biogeographic analysis

Our ancestral area reconstructions (Fig. 1) strongly indicate that the genus *Panolis* originated in South and Central China. Subsequently, the ancestor of Clade A and its descendant species were restricted to the area of origin, whereas a species in Clade B at some point colonized the Palearctic and gave rise to *P. flammea* and *P. japonica*.

## Discussion

### 1. Taxonomy and phylogeny

From a molecular point of view, the mitochondrial *COI* gene (used for “DNA barcoding”) has been considered to be an effective tool for the discrimination of species level taxa in Lepidoptera [37]–[42]. The genetic distances between species in Lepidoptera are usually greater than 3% [37]. In this study, the new species *P. ningshan* **sp. nov.** is well recognized by the minimum distance values of 6.2% from its allied species *P. estheri*. It is also clearly defined morphologically by having a juxta with a long, strongly sclerotized digitate process. Two species, *P. exquisita* and *P. variegatoides*, show a low genetic divergence of 1.9%. Here we temporarily treat them as two distinct species, as *P. exquisita* can be morphologically separated from *P. variegatoides* by having a broader valve and cucullus, as well as stronger thorns on the carina. Moreover, cases of low genetic divergence are also observed among some closely related noctuid species [41].

Taxonomically, we can divide the genus *Panolis* into two species groups: the *P. flammea* species group and the *P. exquisita* species group, supporting the opinions of Ronkay et al. [2], [3]. The *P. exquisita* species group, comprising *P. exquisita*, *P. variegatoides* and *P. pinicortex*, is well characterized by the following traits: basal part of uncus narrow, distal part strongly dilated, triangular; valva broad basally, relatively narrow apically, cucullus somewhat round; posterior part of ductus brusae short sclerotized. The *P. flammea* species group, including *P. flammea*, *P. japonica*, *P. estheri* and *P. ningshan* **sp. nov.**, is well-defined by: uncus narrow basally, slightly dilated apically, valva broad at base, tapering from distal 1/3 to apex, cucullus elongated; ductus brusae long sclerotized at posterior part.

The monophyly of the genus *Panolis* and both the species groups are well supported by our results of phylogenetic analysis with high confidence (Figs. 1–3). Within the *P. exquisita* species group (Clade A) *P. variegatoides* and *P. exquisita* form a sister group, which is close to *P. pinicortex*. In the morphological phylogenetic analysis, the relationships of the *P. flammea* species group (Clade B) are unresolved, due to the small number of morphological characters that can be used for the analysis. However, the analyses of molecular data as well as the combined molecular and morphological data result in more robust and reliable phylogeny reconstructions than using morphological data alone, indicating that *P. estheri* is closer to the sister group *P. flammea* + *P. japonica* than *P. ningshan* **sp. nov.**

Bayesian inference using the Markov Chain Monte Carlo approach provides a powerful alternative for phylogenetic analysis, compared with more traditional maximum parsimony [43]. However, this Bayesian analysis method also remains controversial due to problems such as application of priors [44]–[46] and general skepticism regarding use of model-based approaches [47], [48], In view of these possible problems, it is gratifying to note that we find the same phylogeny for the *Panolis* group regardless of methods.

### 2. Divergence times and biogeographic implications

Although many different mutation rates have been reported for the *COI* gene in insects to date [49], here we used a faster rate of 2.3% per My and a slower rate of 1.5% per My to calibrate the divergence times of the nodes, as they are the most commonly used rates in studies on Lepidopteran insects [50]–[54]. Furthermore, similar rates for the *COI* gene have also been independently found in Lepidoptera in recent studies, such as 1.66–2.9% per My [55], 1.8% per My [56] and 2.3%–3.1% [57] per My. Hence, the use of these two rates should give reasonable age estimates, suggesting that *Panolis* is a young group, probably diverging during the Late Miocene and Pleistocene.

Previous studies suggested that the genus *Panolis* is a Palearctic group [1], [2], while our results of ancestral areas reconstruction reveal that the common ancestor of *Panolis* species occurred in South and Central China, suggesting an origin in the Oriental region. The *P. exquisita* species group has continued to be limited to the ancestral area, whereas representatives of the *P. flammea* species group migrated northward to the Palearctic region. Only two species within *Panolis*, *P. flammea* and *P. japonica*, are present in this northern region. It can be speculated that the Siberian taiga played an important role for the spread into the Palearctic and the current wide distribution of these two species, because both species are reported to feed on conifer species [1], [58], [59], albeit with a wider range of host plants in *P. flammea*. Furthermore, these two species have an allopatric distribution. *P. flammea* is widely distributed in Europe and the west Siberian taiga, with the eastern-most distribution from the Krasnoyarsk area in Russia, whereas *P. japonica* is recorded to be restricted to areas east of the Siberian taiga including Primorye Territory (Russia), northeast China, Korea and Japan. The fauna of the East Siberian taiga is considered to be older than that of the western Siberia taiga, with the Yenisei River as a biogeographic boundary [60]. The ancestral areas reconstruction in this study indicates that the common ancestor of *P. flammea* and *P. japonica* may have occurred in the East Palearctic with the ancestor of *P. flammea* later dispersing to the West Palearctic, but it is also possible that the common ancestor had a very wide Palearctic distribution across the taiga and later split into a western and eastern species.

In conclusion, it can be noted that *Panolis* has no members in the New World today, but the history of the genus suggests a clear possibility for a representative of the Palearctic clade to become established as an invasive species in the Nearctic taiga, for instance following a human-aided accidental introduction. In this case there is a risk that it could become a pest on native pine species, but experience from *P. flammea* in Britain point to the possibility for control by generalist natural enemies after an initial period in an “enemy-free space” [61].

### 3. Systematics

Genus *Panolis* Hübner, [1821] *Panolis* Hübner, 1821:214 [6]. Type species: *Noctua flammea* [Denis & Schiffermüller], 1775, designated by Hampson, 1905: 461 [7]. Synonyms: *Ilarus* Boisduval 1829 *Hilarus* Agassiz [1847] Diagnosis. Medium-sized. Compound eyes relatively small, hairy. Proboscis well developed. Labial palpus short, protrudent, covered with long hairs. Male antenna filiform, with fasciculate cilia. Forewing narrow, elongate, ground color reddish, often with well-defined orbicular and reniform stigmata. Uncus short, basal half narrow, apical half dilated, spatulate, sometimes triangular. Valva elongated, tapering distally, cucullus, rounded or acute, corona absent. Ostium bursae broad, sclerotized. Ductus bursae often ribbed.

**Figure 4:**
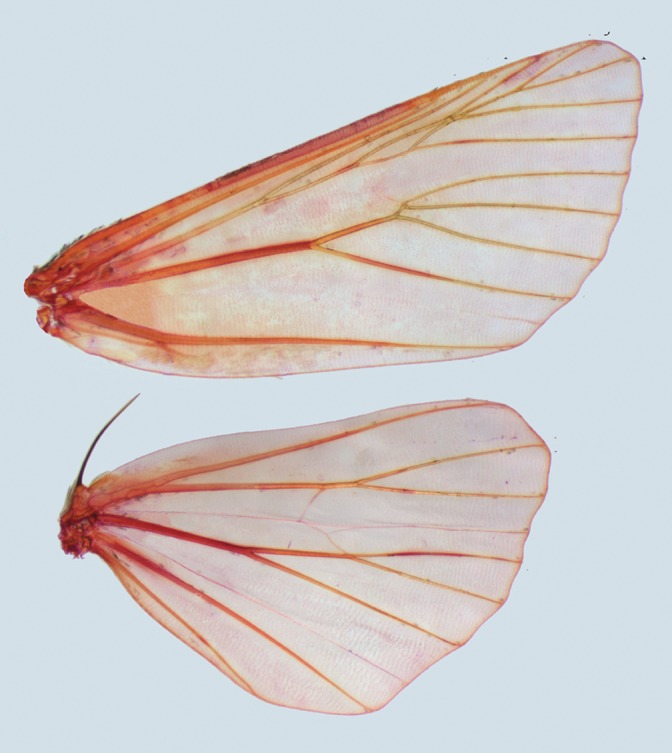
Wing venation of the genus *Panolis*. *P. flammea*, male.

**Figure 5:**
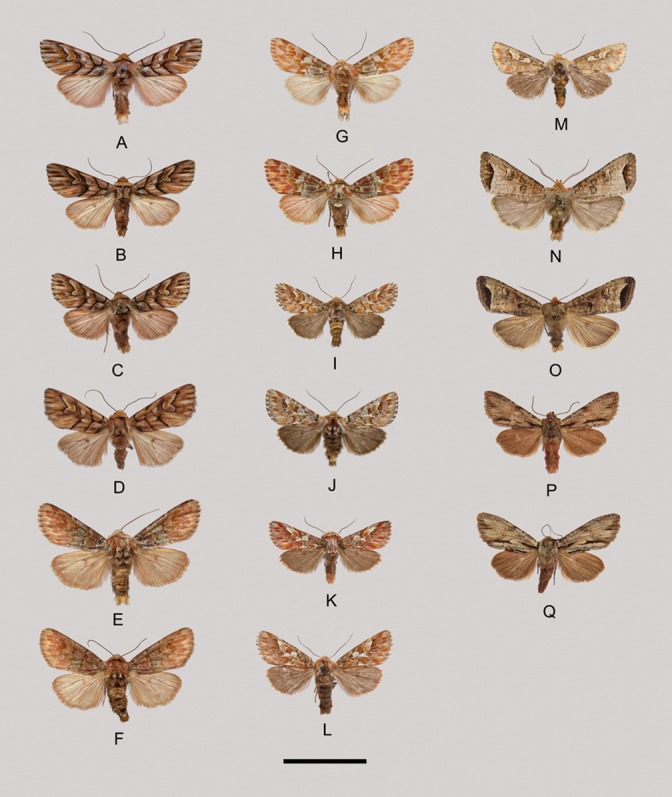
Adults. A–B. *Panolis exquisita*, A. male, B. female; C–D. *P. variegatoides*, C. male, D. female; E–F. *P. pinicortex*, E. male, F. female; G–H. *P. ningshan* **sp.nov.**, G. male, holotype, H. female, paratype; I–J. *P. estheri*, I. male, J. female; K–L. *P. japonica*, K. male, L. female; M. *P. flammea*, male; N–O. *Pseudopanolis heterogyna*, N. male, O. female; P–Q. *Egira acronyctoides*, P. male, Q. female. Scale bar = 1 cm.

Description (Figs. 4–5). Forewing length 13–17 mm. Head small. Frons and vertex with dense hairs. Compound eyes relatively small. Labial palpus short, protrudent, covered with long hairs. Proboscis well developed. Antenna filiform, with fasciculate cilia on male. Thorax robust. Tegulae well-developed. Forewing narrow, elongated, outer margin evenly arcuate, orbicular stigma present, often clearly defined, reniform stigma evidently large; venation with R1 arising from near middle of cell, R2 from apex or near apex of areole, R3, R4 and R5 from apex of areole, R3 stalked with R4, M1 branching from upper angle of cell, M2, M3 and Cula from under angle of cell, respectively, Culb originating from beyond middle of cell, parallel to Cula. Hindwing short, wider than forewing, apex rounded; venation with Sc+R1 arising from near base of cell, Rs and M1 originating separately from upper angle of cell, or short stalked, M2 absent, M3 and Cula branching from under angle of cell, Cu1a almost parallel to Cu1b. Abdomen with a small dorsal tuft on the second segment.

**Figure 6:**
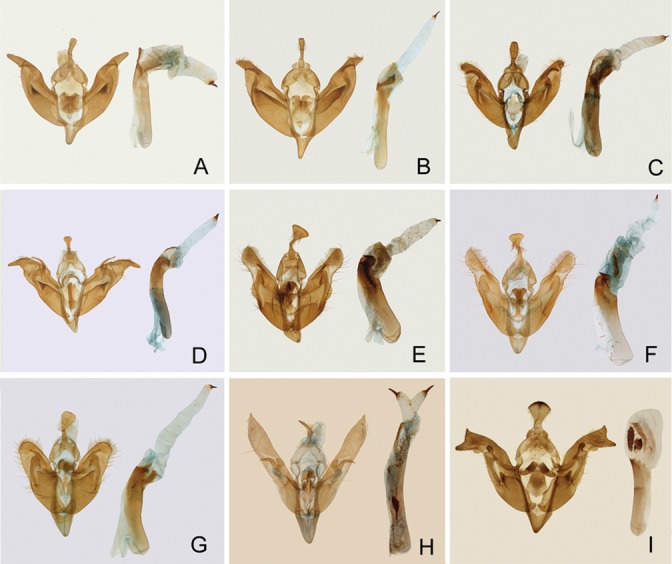
Male genitalia. A. Panolis flammea; B. P. japonica; C. P. estheri; D. P. ningshan **sp. nov.**; E. P. exquisita; F. P. variegatoides; G. P. pinicortex; H. Egira acronyctoides; I. Pseudopanolis heterogyna.

Male genitalia (Fig. 6). Uncus short, basal half narrow, apical half dilated, spatulate, sometimes triangular. Tegumen relatively narrow, elongated. Vinculum rather short, sclerotized. Valva elongated, tapering distally, cucullus rounded or acute, corona absent; ampulla well-developed, digitate or falcate; harpe absent or present. Sacculus with often sclerotized dorso-apical part. Saccus small. Aedeagus cylindrical, somewhat curved distally, carina with one or two large lateral plates, vesica tubular, broad basally, sometimes with a row of small spinules, apex with a sclerotized terminal cornuti.

**Figure 7:**
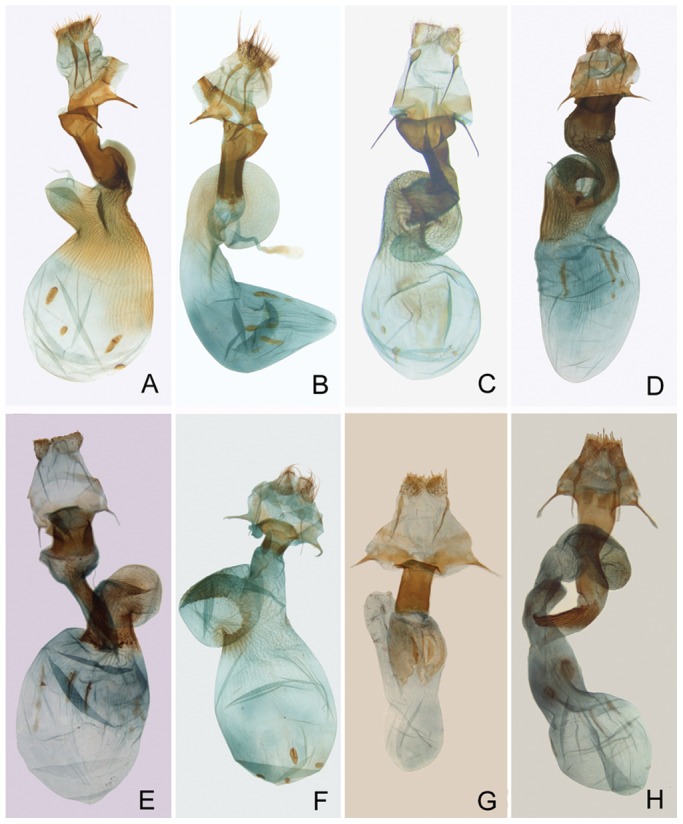
Female genitalia. A. P. japonica; B. P. estheri; C. P. ningshan **sp. nov.**; D. P. exquisita; E. P. variegatoides; F. P. pinicortex; G. Pseudopanolis heterogyna; H. Egira acronyctoides.

Female genitalia (Fig. 7). Ovipositor short, weak. Anal papillae small, densely setose. Gonapophyses short or medium-long. Ostium bursae broad, sclerotized, calyculate, trapezoidal or subdeltoidal. Ductus bursae flattened, posterior part sclerotized, anterior part membranous, often ribbed. Appendix bursae membranous, helicoid, subconical, with ribs. Corpus bursae elliptic or conical, sometimes ribbed, with two or four signum-stripes.

Remarks. The genus was formerly assigned to the tribe Ipimorphini by Beck [62], only based on certain larval characters. Ronkay et al. [2] transferred the genus to the tribe Orthosiini and divided it into two species-groups: the *flammea* species-group and the *exquisita* species-group.

Host plants of this genus are poorly investigated, only two species, *P. flammea* and *P. japonica*, have been reported feeding on conifers such as *Pinus* species [1], [58].

Key to *Panolis* species based on male genitalia

1 Juxta with a long digitate process…………*P. ningshan* **sp. nov.**

Juxta without a long digitate process….………………………2

2 Uncus triangular in distal half…………………………………3

Uncus clavate, not triangular in distal half……………………5

3 Aedeagus without stronger thorns on the carina……*P. pinicortex*

Aedeagus with stronger thorns on the carina…………………4

4 Valva and cucullus broader………………………… *P. exquisita*

Valva and cucullus narrower…………………….*P. variegatoides*

5 Cucullus less elongate, harpe present……………………*P. estheri*

Cucullus elongate, harpe absent……………….…………….6

6 Vesica wider, cucullus more elongated, less acute apically…………………………………………………………*P. flammea*

–Vesica narrower, cucullus triangular, acute apically…………………………………………………………*P. japonica*

### Panolis flammea species group

*Panolis flammea* ([Denis & Schiffermüller], 1775) (Figs. 5M, 6A)

*Noctua flammea* [Denis & Schiffermüller], 1775: 87 [63]. Type locality: Austria, Vienna region.

*Phalaena griseovariegata* Goeze, 1781:250 [64]. No locality given.

*Phalaena piniperda* Loschege, 1785: 55. No locality given.

*Phalaena pini* de Villers, 1789: 278 [65]. Type locality: France.

*Phalaena telifera* Paykull, 1786: 60 [66]. Type locality: Sweden.

*Bombyx spreta* Fabricius, 1787: 124 [67]. Type locality: Germany.

Diagnosis. The species is very similar to *P. japonica*, but can be easily distinguished by forewing distinctly marked with antemedian and postmedian lines, and relatively smaller, ochreous reniform stigma; Male genitalia with narrower valve, cucullus more elongated, less acute apically, vesica wider, with a thicker terminal cornuti; Female genitalia with ductus bursae longer, sclerotized, proximally less broad, corpus bursae more elongated, with smaller ribbed portion.

Description. Male. Forewing length 14–16 mm. Frons and vertex scattered with white hairs, compound eyes small. Antenna filiform, fasciculate-ciliate. Proboscis well developed. Labial palpus short, covered with long hairs. Thorax red brown, with white scales. Forewing narrow, brownish red suffused, marked with ochreous and white scales, with a conspicuous white orbicular stigma and a larger ochreous reniform stigma, cilia purplish brown; venation with R2, R3, R4 and R5 arising from apex of areole, R3 and R4 stalked about 1/3. Hindwing brown; venation Rs and M1 short stalked. Abdomen yellow gray, dark laterally.

Male genitalia. Uncus short, narrow at basal half, apical half dilated, slightly flattened. Tegumen relatively narrow. Valva elongated, strongly tapering at distal third, cucullus less acute, corona missing, sacculus long, strongly sclerotized at dorsal apical part, ampulla sickle-shaped, harpe absent. Saccus small. Aedeagus cylindrical, somewhat curved distally, carina with large, dentate lateral plate. Vesica broad basally, with many spinules, distal part tubular, with a terminal cornuti.

Female. Very similar to male except simple antenna, and conical terminal of abdomen.

Female genitalia. Anal papillae small, densely setose. Posterior apophysis longer than anterior apophysis. Ostium bursae wide, sclerotized, calyculate. Ductus bursae flattened, posterior part sclerotized, tubular, anterior part membranous, with stronger ribs. Appendix bursae straight, cylindrical, basal half with ribs. Corpus bursae elliptical, caudal part ribbed, with four signum-stripes.

Materials examined. 2♂, Deutschland Brandenburg Ketzur, Germany, 20–30. V. 2010; 1♂, Műnchen-Obermenzing, Bavaria, Germany, 3. IV. 2011, leg. Ulf Buchsbaum.

Distribution. Euro-western Siberian.

Remarks. The species is widely distributed in Europe, extending eastward to Krasnoyarsk area in Russia [1]. It also occurs in Asia Minor and Transcaucasia[2]. Sinev [68] listed a distribution record in the Amur region in the Catalogue of the Lepidoptera of Russia, this record is however highly uncertain, as there is no original literature reporting specimens from the Amur region. The immature stages and biological information of this species were reported by Carter [69] and Molet [58], and the larva is considered as an important pest of some *Pinus* and *Picea* species.

*Panolis japonica* Darudt, 1935 (Figs. 5K–L, 6B, 7A)

*Panolis flammea* form *japonica* Draudt, 1935: 199 [70]. Type locality: Japan, Kobe.

*Panolis flammea* form *sutshana* Draudt, 1935: 199 [70]. Type locality: U.S.S.R. Sutschanski Rudnik.

*Panolis flammea japonica* Inoue & Sugi 1958: 468 [71].

*Panolis japonica* Kononenko & Mikkola, 1989:29 [1].

Diagnosis. This species differs from the closest species *P. flammea* by indistinctly serrated male antenna; forewing more oblong, reniform stigma larger; male genitalia smaller, uncus narrow at basal half, gradually dilated towards apex, sacculus with deep basal invagination, vesica with a more slender terminal cornuti; female genitalia with larger ribbed portion on corpus bursae.

Description: Male. Forewing length 13–15 mm. Frons and vertex covered with orange yellow hairs, compound eyes small. Antenna filiform, indistinctly serrated. Proboscis well developed. Labial palpus short, covered with long pale yellow hairs Thorax red brown, with white hairs. Tegulae grey. Forewing varies from pale reddish to bright reddish, costa with white spot at basal area. Antemedian and postmedian lines obscure or lacking, reniform stigma larger, more falciform, with red brown patch medially. Orbicular stigma white. Cilia pale on veins, reddish brown between veins; venation with R2 arising from near apex of areole, R3 stalked with R4 about 1/3. Hindwing grey brown, cilia pale, with a few reddish brown speckles; venation with Rs and M1 short stalked. Abdomen reddish brown or brown.

Male genitalia. Uncus short, basal half narrow, gradually dilated towards apex, slightly flattened apically. Tegumen relatively narrow. Juxta arrowheaded. Valva elongated, tapering at distal third, cucullus triangular, corona absent, sacculus long, with deep basal invagination, ampulla sickle-shaped, harpe absent. Saccus small, V-shaped. Aedeagus cylindrical, somewhat curved distally, carina with large lateral plate, vesica broad at basal part, with many small spinules, distal part tubular, with a fine terminal cornuti.

Female. Very similar to male.

Female genitalia. Anal papillae small, densely setose. Posterior apophysis longer than anterior apophysis. Ostium bursae broad, calyculate. Ductus bursae flattened, posterior part well sclerotized, tubular, anterior part membranous, with stronger ribs. Appendix bursaes straight, cylindrical, round apically, basal half with ribs. Corpus bursae elliptical, posterior part ribbed, anterior part membranous, with four signum-stripes.

Materials examined. 1 ♂, 2♀, Horoman, Samani, Hokkaido, Japan, 20. V. 1995, leg. Hiroyuki Kogi, 1 ♂, 1♀, same locality, 5. V. 2000, leg. Hiroyuki Kogi; 1♀, Shiozawa spa, Fukushima Pref., Japan, 3. V. 1968, leg. Toshiya Ebato; 4 ♂, 2♀, Kawaburu spa, Gumna Pref., Japan, 5. V. 1967, leg. Toshiya Ebato; 5 ♂, 2♀, Omeitei, Nasu Imperial Villa, Tochigi Pref., Japan, 27–29. IV. 2006, leg. Yutaka Arita, Mamoru Owada & Koji Yasuda; 1♀, Mt. Mitsumine, Saitama Pref., Japan, 5. V. 1959, leg. Toshiya Ebato; 1♀, Mt. Takaosan, Tokyo, Japan, 15. IV. 1964, leg. Mamoru Owada; 1 ♂, same locality, 17. IV. 1976, leg. Hiroshi Yoshimoto; 1♂, Kiyose, Tokyo, Japan, 17. IV. 1958, leg. Toshiya Ebato; 1♂, Odarumi-toge, Kanagawa Pref., 15. III. 1979, leg. Mamoru Owada; 1♂, same locality, 24. III. 1979, leg. Hiroshi Yoshimoto; 1♀, same locality, 31. III. 1979, leg. Hiroshi Yoshimoto; 1♀, same locality, 7. IV. 1979, leg. Hiroshi Yoshimoto; 1♂, same locality, 16. IV. 1977, leg. Hiroshi Yoshimito; 1♀, same locality, 23. IV. 1977, leg. Hiroshi Yoshimoto; 1♂, Sasago, Yamanashi Pref., 7. IV. 1979, leg. Hiroshi Yoshimoto; 2♂, Saiko Lake, Fuji-Kawaguchiko, Yamanashi Pref., Japan, 11. IV. 2009, leg. Yasunori Kishida; 4♂, 1♀, Kurechi, Yamanashi Pref., Japan, 16. IV. 1977, leg. Hiroshi Yoshimoto; 1♂, Fukashiro, Yamanashi Pref., Japan, 10. IV. 1977, leg. Hiroshi Yoshimoto; 1♀, same locality, 16. IV. 1977, leg. Hiroshi Yoshimoto; 1♂, 1♀, same locality, 24. IV. 1976, leg. Hiroshi Yoshimoto; 1♀, Yabunoyu spa, Yamanashi Pref., Japan, 1. V. 1971, leg. Toshiya Ebato; 4♂, 1♀, same locality, 4. V. 1970, leg. Toshiya Ebato; 1♀, same locality, 5. V. 1972, leg. Toshiya Ebato; 1♀, same locality, 15. V. 1971, leg. Toshiya Ebato; 1♂, Uenohara, Yamanashi Pref., Japan, 2. IV. 1977, leg. Hiroshi Yoshimoto; 1♂, same locality, 7. IV. 1979, leg. Hiroshi Yoshimoto; 1♂, same locality, 23. IV. 1977, leg. Hiroshi Yoshimoto; 1♀, Sagashio, Yamanashi Pref., Japan, 15. IV. 2001, leg. Hideki Kobayashi; 2♀, Shimashima, Nagano Pref., Japan, 4. V. 1969, leg. Toshiya Ebato; 1♂, Nyuyama, Nagawa, Nagano Pref., Japan, 4. V. 1999, leg., Mamoru Owada; 1♀, Kishojihara, Nagawa, Nagano Pref., Japan, 14. V. 1994, leg. Mamoru Owada; 1♂, Hotaka, Azumino, Nagano Pref., Japan, 27. IV. 2008, leg. Hideki Kobayashi; 1♂, Shirakabako, Azumi, Nagano Oref., Japan, 10. V. 1990, leg. Hideki Kobayashi; 1♂, 2♀, Iida, Nagano Pref., Japan, 21. IV. 1993, leg. Hedeki Kobayashi; 1♂, Kizakiko, Ohmachi-city, Pref. Nagano, Japan, 1. V. 2011, leg. Yoshihiro Yanagita; 2♂, 1♀, Akechigahara, Shimojo, Nagano Pref., Japan, 7. IV. 2011, leg Hiroshi Endo; 5♂, 4♀, Akashina, Nagano Pref., Japan, 1–5. V. 2011, leg. Yoshihiro Yanagita & Yasunori Kishida; 3♂, 2♀, Takayama, Gifu Pref., Japan, 23–27. IV. 1977, leg. Satoshi Hashimoto; 1♂, 1♀, Nomugi-toge, Gifu & Nagano Pref., Japan, 20–21. V. 1988, leg. Mamoru Owada; 2♂, R. Otogawa, Hongu, Wakayama Pref., Japan, 22. III. 1995, leg. Mamoru Owada; 1♂, Kotonotaki, Susami, Wakayama Pref., Japan, 23. III. 1995, leg. Mamoru Owada; 1♂, Naidaijin, 600 m in alt., Kumamoto Pref., Japan, 6. IV. 2002, leg. Hideki Kobayashi; 1♂, 1♀, same locality, but 900 m in alt., 8. III. 2002, leg. Hideki Kobayashi; 3♂, 2♀, Tankaino, Itsuki, Kumamoto Pref., Japan, 13. IV. 2003, leg. Hideki Kobayashi; 1♂, same locality, 7. IV. 2003, leg. Hideki Kobayashi; 1♀, Nihonsugi, Izumi, Kumamoto Pref., Japan, 9. V. 2002, leg. Hideki Kobayashi; 1♂, Kamitsushima, Tsushima, Japan, 28. III. 2012, leg. Katsumi Shibahara; 1♂, 1♀, Omasu, Kamitsushima, Tushima, Japan, 10. III. 2013, leg. Mamoru Owada; 4♂, Kuchinashidani Tunnel, Kamitsushima, Tsushima, Japan, 11. III. 2013, leg. Mamoru Owada.

Distribution. China (Heilongjiang), Japan, Korea, Russia (Primorye Territory).

Remarks: The larva of this species was illustrated by Yamamoto [59], and it also feeds on *Pinus* species.

*Panolis estheri* Ronkay, Ronkay, Gyulai & Hacker, 2010 (Figs. 5I–J, 6C, 7B)

*Panolis estheri* Ronkay, Ronkay, Gyulai & Hacker, 2010: 128 [3]. Type locality: China, Shaanxi.

Diagnosis. This species resembles *P. flammea* and *P. japonica*, but can be distinguished by reniform stigma on the forewing less elongated, white-gray, with orange yellow patch medially; hindwing darker; male genitalia with valve less enlongated distally, harpe present, cucullus rounded; female genitalia with appendix bursae longer, twisted, weakly ribbed, corpus bursae curved, conical.

Description: Male. Forewing length 13 mm. Frons and vertex covered with orange yellow hairs, compound eyes small. Antenna filiform, fasciculate-ciliate. Proboscis well developed. Labial palpus short, with long yellow brown hairs. Thorax blood red, with white hairs. Tegulae mixed with yellow brown, blood red and white hairs. Forewing yellow brown, with ash-grey scales, especially in basal area, reniform stigma less elongated, white-gray, with orange yellow patch medially, orbicular stigma ash-grey, a remarkable red brown quadrangular patch between the orbicular and reniform stigma. Postmedial and subterminal lines red brown, zigzagged. Cilia red brown at basal half of veins, black at the distal half, white between the veins; venation with R2, R3, R4 and R5 arising from apex of areole, R3 stalked with R4 about 1/2. Hindwing darker; venation with Rs and M1 short stalked. Abdomen dark brown.

Male genitalia. Uncus short, narrow at basal 1/3, dilated at apical 2/3, flattened apically. Tegumen relatively narrow. Valva less elongated distally, tapering at distal third, cucullus slightly round, corona absent, sacculus long, ampulla strongly sclerotized, curved medially, harpe present, broad basally, tapering toward apex, pointed apically. Saccus small, V-shaped. Aedeagus cylindrical, somewhat curved distally, carina with large sclerotized lateral plate, vesica broad basally, distal part tubular, with a fine terminal cornuti.

Female. Similar to male except the larger size.

Female genitalia. Anal papillae small, densely setose. Posterior apophysis longer than anterior apophysis. Ostium bursae broad. Ductus bursae short, posterior part sclerotized, tubular anterior part less ribbed. Appendix bursae longer, twisted, with weakly ribs. Corpus bursae boot-shaped, more conical at posterior part, round apically, with four signum-stripes.

Materials examined. 1 ♂, 1♀, Jialingjiangyuantou, Baoji, Shaanxi, China, 18. IV. 2012, leg. Mamoru Owada & Min Wang.

Distribution. China (Shaanxi).

Remarks: The male adult was described here for the first time.

*Panolis ningshan* Wang, Fan, Owada, Wang & Nylin **sp. nov.** urn:lsid:zoobank.org:act:EC2F4509-73EC-4068-9759-368C93E4C96E (Figs. 5G–H, 6D, 7C)

Diagnosis: The new species can be easily separable from the other three species of the *P. flammea* group by hindwing somewhat dark at basal 1/3, pale reddish-ochreous at apical 2/3; male genitalia with apical area of uncus more dilated, juxta with a long, strongly sclerotized digitate process;female genitalia with a larger ostium bursae, appendix bursae twisted, ribbed.

Description: Male. Forewing length 15–17 mm. Frons and vertex white, with red-brown hairs, compound eyes small. Antenna filiform, with densely white cilia. Proboscis well developed. Labial palpus short, covered with long dark brown hairs. Thorax and tegulae red-brown, with white hairs. Forewing deep ash-grey at basal area, with a dark brown patch on costa. Antemedial, postmedial and subterminal lines blood red, orbicular stigma white, reniform stigma brown, encircled with white scales, cilia blood red on veins, white between the veins; venation with R2, R3, R4 and R5 from apex of areole, R3 stalked with R4 stalked about 1/2. Hindwing slightly dark at basal 1/3, pale reddish-ochreous at apical 2/3, cilia pale yellow; venation with Rs and M1 short stalked. Abdomen yellow brown.

Male genitalia. Uncus short, basal part narrow, apical part dilated, spatulate, flattened apically. Tegumen narrow. Valva elongated, tapering from middle to apex, cucullus digitate, corona absent, sacculus long and broad, ampulla long, curved near base, harpe present, slender, clavate. Saccus small, V-shaped. Aedeagus cylindrical, somewhat curved distally, carina with a sclerotized lateral plate, vesica broad at basal part, with a sclerotized cornuti stripe, distal part tubular, with a terminal cornuti.

Female. Similar to male.

Female genitalia. Anal papillae small, densely setose. Gonapophyses slender, posterior apophysis almost as long as anterior apophysis. Ostium bursae enlarged. Ductus bursae short, posterior part tubular, sclerotized, curve. Appendix bursae long, twisted, ribbed. Corpus bursae somewhat round, with four signum-stripes.

Materials examined. Holotype: ♂, Guanghuojie, Ningshan, Shaanxi, China, 22. IV. 2012, leg. Min Wang & Mamoru Owada; Paratypes: 3 ♂, 1♀, same data as the holotype; 6♂, 2♀, same locality with the holotype, 4–8. V. 2011, leg. Houshuai Wang. 4♂, Wuyishan, Jiangxi, China, 2. IV. 2012, leg. Houshuai Wang & Haiming Xu; 4♂, Wuyishan, Jiangxi, China, 21. V. 2011, leg. Houshuai Wang & Xiaohua Deng; 7♂, Jiwozi, Xian, Shaanxi, China, 21–22. IV. 2012, leg. Mamoru Owada & Min Wang.

Distribution. China (Shaanxi, Jiangxi).

Etymology: The species is named after its type locality.

### Panolis exquisita species group

*Panolis exquisita* Draudt, 1950 (Figs. 5A–B, 6E, 7D)

*Panolis exquisita* Draudt, 1950: 45 [8]. Type locality: China, Zhejiang, Tian-mu-shan.

Diagnosis: *P. exquisita* Drauat is closely related to *P. variegatoides* Poole, but can be easily separated from the latter by paler forewing and hindwing; male genitalia with broader valve and cucullus, wider and stronger thorns on the carina; female genitalia with more subdeltoidal ostium bursae, posterior part of ductus bursae broader, strongly sclerotized.

Description. Male. Forewing length 14–17 mm. Frons and vertex covered with pale yellow hairs. Antenna filiform, fasciculate-ciliate. Proboscis well developed. Labial palpus short, outer margin yellow brown, inner margin pale yellow. Thorax orange brown, sometimes with dark hairs. Tegulae orange brown. Forewing varies from orange yellow to dark brown, basal area with a black Y-shaped antemedial line, a black medial line from middle of dorsum to cell, L-shaped, two pale yellow elliptical stigma between two lines, reniform stigma large, falciform, pale yellow, with orange brown patch medially, subterminal and terminal area with short black longitudinal stripes between veins; venation with R2, R3, R4 and R5 arising from apex of areole, R3 stalked with R4 about 1/2. Hindwing pale yellow, with a dark dot at end of cell; venation with Rs and M1 stalked very shortly. Abdomen orange brown, terminal with pale yellow tufts.

Male genitalia. Uncus short, basal half slender, distal half dilated, triangular, apical somewhat arched. Tegumen narrow. Vinculum short. Saccus small. Valva elongated, tapering toward apex, cucullus rounded, setose, corona absent, sacculus short, clavus reduced, harpe broad, apex rounded; ampulla straight, apically tapering. Aedeagus cylindrical, distal part somewhat curved, carina with strong thorns. Vesica tubular, basal part with a long cornuti stripe consisting of small spinules, distal part with a terminal cornutus.

Female. Very similar to male.

Female Genitalia. Anal papillae small, densely setose. Posterior apophysis twice longer than anterior apophysis. Ostium bursae subdeltoidal, sclerotized. Posterior part of ductus bursae broad, sclerotized, anterior part tubular, with sclerotized ribs. Appendix bursae conical, apically twisted, strongly ribbed. Corpus bursae elliptical, with three signum-stripes.

Materials examined. 17 ♂, Nanling National Nature Reserve, Guangdong, China, 29. III-1. IV. 2003, leg. Mamoru Owada, Yasunori Kishida & Min Wang; 4♂, 2♀, same locality, 23–25. IV. 2004, leg. Horie Kiyoshi & Min Wang; 3♂, 6♀, same locality, 11–18. V. 2006, leg. Mamoru Owada & Min Wang; 2♂, 7♀, same locality, 16–20. V. 2009, leg. Mamoru Owada & Min Wang; 3♂, same locality, 8. V. 2011, leg. Mamoru Owada & Min Wang; 3♂, 2♀, Guanghuojie, Ningshan, Shaanxi, China, 5–8. V. 2011, leg. Houshuai Wang. 2♂, same locality, 22. IV. 2012, leg. Mamoru Owada & Min Wang; 12♂, Jialingjiang Headwaters, Baoji, Shaanxi, China, 18. IV. 2012, leg. Mamoru Owada & Min Wang; 34♂, 2♀, Hotel, Jiwozi, Xian, Shaanxi, China, 21–22. IV. 2012, leg. Mamoru Owada & Min Wang; 3♂, Wuyishan, Jiangxi, China, 21. V. 2011, leg. Houshuai Wang.

Distribution. China (Shaanxi, Zhejiang, Jiangxi, Fujian, Guangdong, Yunnan)

Remarks. This species is widely distributed from South to Central China [8], [72], [73]. The following moth is the sister species.

Panolis variegatoides Poole, 1989 (Figs. 5C–D, 6F, 7E)

*Hadena variegatoides* Poole, 1989: 481 [9], replacement name for *Hadena variegata* Wileman, 1914. Type locality: China, Formosa [Taiwan], Rantaizan.

*Hadena variegata* Wileman, 1914: 162 [74], nec *Hadena variegata* Staudinger, 1871.

*Panolis exguisita*: Sugi, 1992: 199 [75], misidentification.

*Panolis variegatoides* Hreblay & Ronkay, 1997:30 [76]

Diagnosis: The species is superficially similar to the preceding species, but can be distinguished by male genitalia with narrower valva and cucullus, carina with much smaller thorns; female genitalia with rounded ostium bursae, posterior part of ductus bursae broadened, with weakly sclerotized swollen area.

Description. Male. Forewing length 15–16 mm. Frons and vertex covered with pale yellow hairs. Antenna filiform, fasciculate-ciliate. Proboscis well developed. Labial palpus short, outer margin yellow brown, inner margin pale yellow. Thorax reddish brown. Tegulae reddish brown. Forewing reddish brown, antemedial line black, Y-shaped, medial line black, short, L-shaped, two pale yellow elliptical stigma between two lines, reniform stigma large, falciform, pale yellow, with orange brown patch medially, subterminal and terminal area with short black longitudinal stripes between veins; venation with R2 arising from near apex of areole, R3 stalked with R4 about 1/2. Hindwing pale yellow, with a dark dot at end of discal area; venation with Rs and M1 short stalked. Abdomen orange brown, terminal with pale yellow tufts.

Male genitalia. Uncus short, basal half slender, distal half dilated, triangular, apical somewhat arched. Tegumen narrow. Vinculum short. Saccus small. Valva relatively narrow, elongated, tapering toward apex, cucullus narrow, rounded, setose, corona absent, sacculus short, clavus reduced, harpe wide, apex rounded, ampulla straight, apically tapering. Aedeagus cylindrical, distal part somewhat curved, carina with clearly thorns. Vesica tubular, basal part with a long cornuti stripe consisting of small spinules, distal part with a terminal cornuti.

Female. Very similar to male.

Female Genitalia. Anal papillae small, densely setose. Posterior apophysis twice longer than anterior apophysis. Ostium bursae slightly rounded, sclerotized. Posterior part of ductus bursae broad, with weakly sclerotized swollen area, anterior part tubular, narrow, with sclerotized ribs. Appendix bursae conical, apically twisted, ribbed. Corpus bursae elliptical, with three signum-stripes.

Materials examined. 1♂, Tayuling, Hualien, Taiwan, China, 24–25. III. 1980, leg. Toru Shimomura; 1♂, Lushan spa, Nantou, Taiwan, China, 29. III. 1980, leg. Tatsuya Tanabe; 1♂, 2♀, same locality, 8. IV. 1980, leg. Toru Shimomura; 29♂, 3♀, Chun Yang, Nantou, Taiwan, China, 25–31. III. 1981, leg. Hiroshi Yoshimoto; 12♂, 5♀, same locality, 23–24. III. 1982, leg. Hiroshi Yoshimoto; 1♂, Anmashan, Tahsuehshan Mts., Taichung, Taiwan, China, 14–16. VI. 1993, leg. Mamoru Owada; 5♂, Pilu-Shenmu, Hualien, Taiwan, China, 14. III. 2010, leg. Mamoru Owada; 3♂, Chinma Tunnel, Hualian, Taiwan, China, 17. III. 2012, leg. Mamoru Owada, Shipher Wu & Wei-Chun Chang; 1♂, Meifeng, Nantou, Taiwan, China, 19. III. 2012, leg. Mamoru Owada; 1♂, 1♀, same locality, 8. V. 2013, leg. Mamoru Owada, Li-Cheng Shih & Yen-Lin Chen. 3♀, Tzuen, Hualien, Taiwan, 7. V. 2013, leg. Mamoru Owada & Shipher Wu; 3♂, 2♀, Kuanyuan, Hualien, Taiwan, 7. V. 2013, leg. Mamoru Owada & Shiper Wu.

Distribution. China (Taiwan).

Remarks. This species is endemic to Taiwan.

*Panolis pinicortex* Draudt, 1950 (Fig. 5E–F, 6G, 7F)

*Panolis pinicortex* Draudt, 1950: 45 [8]. Type locality: China, Hunan, Heng Shan, Yunnan, Li Jiang.

*Panolis pinicortex exornata* Hreblay & Ronkay, 1997: 28 [76]. Type locality: China, Taiwan, Miaoli.

Diagnosis: The species is superficially similar to the members of *P. flammea* group, especially for the new species *P. ningshan* **sp.nov.**, but can be easily separated from them by male genitalia with distal half of uncus more dilated, triangular, valve less elongated distally, cucullus rounded, harpe present, broad. It also has similar male genitalia characters as *P. exquisita*, but can be distinguished by forewing without black lines, reniform stigma reddish brown, more obscure, valve shorter and wider, aedeagus without strong thorns on carina, female genitalia with posterior part of ductus bursae shortly sclerotized.

Description: Male. Forewing length 16–17 mm. Frons and vertex covered with pale white hairs. Antenna filiform, with fine cilia. Proboscis well developed. Labial palpus short, outer margin brick-red, inner margin pale white. Thorax robust, reddish brown. Tegulae brick-red, marked with brown and whitish grey. Forewing narrow, elongated, apex somewhat rounded, ground colour brick-red, basal area with pale whitish grey irroration, orbicular stigma pale grey, reniform stigma falciform, brick-red, both stigma encircled with whitish scales; venation with R2 arising from near apex of areole, R3 stalked with R4 about 1/2. Hindwing pale reddish-ochreous, with brick-red discal spot, cilia white; venation with Rs and M1 stalked very shortly. Abdomen reddish-ochreous.

Male genitalia: Uncus short, basal part narrow, distal part spatulate, triangular, apical arched. Tegumen narrow. Vinculum V-shaped. Valva broad, slightly tapering to apex; cucullus rounded, setose, corona absent, sacculus strong, rather short, distal end forming a short process, clavus well-developed, rounded, harpe broad, ampulla slightly curved medially, stick-like. Aedeagus cylindrical, curved medially. Vesica long, tubular, with a small acute cornuti.

Female. Very similar to male.

Female genitalia. Anal papillae small, densely setose. Posterior apophysis slender, twice longer than anterior apophysis. Ovipositor short, weak. Ostium bursae trapezoidal with arcuate caudal sclerotized lateral margins. Posterior part of ductus bursae well sclerotized, short, anterior part membranous, ribbed. Appendix bursae broad, twisted, proximal part ribbed. Corpus bursae elliptical, with four short signum-stripes.

Materials examined. 6♂, 2♀, Nanlin National Nature Reserve, Shaoguan, Guangdong, China, 20–24. II. 2003, leg. Mamoru Owada, Katsumi Yazaki, Koji Suzuki & Min Wang; 10♂, 1♀, same locality, 11–14. III. 2004, leg. Mamoru Owada & Min Wang; 1♀, Maoershan, Guilin, Guangxi, China, 26–29. III. 2005, leg. Mamoru Owada & Min Wang; 1♀, Lushan spa, Nantou, Taiwan, China, 23–24. III. 1981, leg. Hiroshi Yoshimoto; 1♂, 1♀, Hsitsun, Taoyuan, Taiwan, China, 24. II. 2009, leg. Mamoru Owada; 1♀, Shengkuang, Taichung, Taiwan, China, 28. II. 2009, leg. Mamoru Owada; 2♂, Hsitsun, Taoyuan, Taiwan, China, 12. III. 2010, leg. Mamoru Owada; 1♂, Yilan, Taiwan, China, 16. III. 2012, leg. S. Wu & W. Chang.

Distribution. China (Anhui, Taiwan, Hunan, Guangdong, Guangxi, Yunnan).

Remarks. This species is widely distributed from South to Central China [8], [72], [73]. Hreblay & Ronkay [76] described Taiwanese population as a separate subspecies.

## Supporting Information

Appendix S1 **Characters and Character states employed in the morphological phylogenetic analysis.**
(DOCX)

Table S1 **Sampling data used in the molecular phylogenetic analyses.**
(DOCX)

Table S2 **Character matrix used in the morphological phylogenetic analysis.**
(DOCX)

Table S3 **Pairwise Kimura 2-Parameter (K2P) distances of** ***COI*** **sequences in all taxa used in this study.**
(DOCX)

Table S4 **The models and model parameters estimated with jModelTest.**
(DOCX)
