# Supplementary material for: Phylogeny, Systematics and Biogeography of the Genus Panolis (Lepidoptera: Noctuidae) Based on Morphological and Molecular Evidence
Source: PLoS One. 2014 Mar 6;9(3):e90598. doi: 10.1371/journal.pone.0090598 (PMC3946178; doi:10.1371/journal.pone.0090598)
Supplement: Appendix S1 — Characters and Character states employed in the morphological phylogenetic analysis. (DOCX) [file pone.0090598.s001.docx]

**Appendix S1**

Characters and Character states employed in the morphological phylogenetic analysis. Character states were scored 0-3 (0= plesiomorphic state, 1-3 = apomorphic state).

1. Antenna: (0) filiform in male and female; (1) dentate in male, filiform in female. In *Pseudopanolis heterogyna* the antenna is dentate in male, filiform in female, while in *Egira acronyctoides* and all *Panolis* species the antenna is filiform in both sexes.
2. Orbicular and reniform stigma on the forewing: (0) absent; (1) present. *P. heterogyna* and all *Panolis* species have distinct orbicular and reniform stigma on the forewing, but in *E. acronyctoides* these stigma are absent.
3. Claviform stigma on the forewing: (0) absent; (1) present. The claviform stigma presents in *P. heterogyna* and *P.* *exquisita* species group*, P. variegatoides* and *P. pinicortex*, but is absent in *E. acronyctoides* and *P. flammea* species group.
4. Black antemedial and medial lines on the forewing: (0) absent; (1) present. *P.* *exquisita* and *P. variegatoides* have distinct black antemedial and medial lines on the forewing, while in the two outgroups and the other *Panolis* species the two lines are not black, often indistinct.
5. R3 and R4 venation: (0) stalked about 1/2; (1) stalked about 1/3. In *P. flammea* and *P. japonica* R3 stalked with R4 about 1/3 while in the two outgroups and the other *Panolis* species R3 and R4 stalked about 1/2.
6. Hindwing color: (0) deep; (1) light, but basal area slightly deep; (2) light. In the two outgroups, *P. flammea, P. japonica* and *P. estheri* the hingwing color is deep, often gray or dark, in *P. ningshan* **sp.nov.** the hindwing is pale reddish-ochreous, with slightly dark at basal area, while in *P. exquisita, P.* *variegatoides* and *P. pinicortex* the hindwing is light, often pale yellow or pale reddish-ochreous.
7. Rs and M1 venation: (0) arising from upper angle of cell; (1) short stalked. In two outroups Rs and M1 arise from upper angle of cell, but in all *Panolis* species they are shortly stalked.
8. Uncus: (0) slender, tapering to apex, apex point; (1) narrow basally, slightly dilated apically; (2) narrow basally, strongly dilated apically. The uncus is slender and tapers to apex in *E. acronyctoides*, it is narrow basally and slightly dilated apically in *P. flammea* species group but strongly dilated apically in *P. heterogyna* and *P. exquisita* species group.
9. Valva: (0) straight, narrow, cucullus pointed; (1) curved distally, cucullus concave medially; (2) broad basally, narrow distally, cucullus somewhat round; (3) broad basally, tapering from distal 1/3 to apex, cucullus elongated. In *E. acronyctoides* the valva is narrow and straight, with pointed cucullus. In *P. heterogyna* it is relatively broader and curved at distal area, and the cucullus concave medially. In *P. exquisita* species group it is broad basally, narrow distally and the cucullus slightly round. While in *P. flammea* species group it is broad basally and tapers from distal 1/3 to apex, with elongated cucullus.
10. Harpe: (0) present; (1) absent. The harpe presents in all examined species except *P. flammea* and *P. japonica*.
11. Ampulla: (0) short, round apically; (1) long, acute apically. In two outgroups the ampulla is short and round apically, while in all *Panolis* species it is long and acute apically.
12. Sacculus: (0) short; (1) long. In the two outgroups and *P. exquisita* species group the sacculus is short, but in *P. flammea* species group it is long.
13. Digitate projection on juxta: (0) absent; (1) present. The digitate projection on juxta is absent in all examined species except the new species *P. ningshan* **sp. nov.**
14. Carina: (0) without strongly sclerotized thorns; (1) with strongly sclerotized thorns. The carina lack strongly sclerotized the thorns in all examined species except *P. exquisita* and *P. variegatoides*.
15. Posterior part of ductus brusae: (0) long sclerotized; (1) short sclerotized. In the two outgroups and *P. flammea* species group the posterior part of ductus brusae is long sclerotized, while it is short sclerotized in *P. exquisita* species group.
16. Corpus bursae (0) elliptical; (1) boot-shaped. In *P. estheri* the corpus bursae is boot-shaped and more conical at anterior part, while in all other examined species it is elliptical.
17. The sclerotized ribs on Posterior part of corpus bursae: (0) absent; (1) present. The Posterior part of corpus bursae lack sclerotized ribs in all examined species except *P. flammea* and *P. japonica*.
18. Signum: (0) absent; (1) present. The signum is absent in two outgroups, while it presents in all *Panolis* species.
