# Supplementary material for: Phylogeny, Systematics and Biogeography of the Genus Panolis (Lepidoptera: Noctuidae) Based on Morphological and Molecular Evidence
Source: PLoS One. 2014 Mar 6;9(3):e90598. doi: 10.1371/journal.pone.0090598 (PMC3946178; doi:10.1371/journal.pone.0090598)
Supplement: Table S1 — Sampling data used in the molecular phylogenetic analyses. (DOCX) [file pone.0090598.s002.docx]

Table S1. Sampling data used in the molecular phylogenetic analyses

| Specimen  voucher no | Taxa | Collection locality | Collection date | Genebank Accession  no (COI) | Genebank Accession  no (16S) | Genebank Accession  no (EF1-a) |
| --- | --- | --- | --- | --- | --- | --- |
| H83 | *Panolis flammea* | Műnchen-Obermenzing, Bavaria, Germany | 03-IV-2011 | KJ194538 | KJ194549 | KJ194560 |
| H100 | *Panolis flammea* | Deutschland Brandenburg, Ketzur, Germany | 20-30-V-2010 | KJ194539 | KJ194550 | KJ194561 |
| H101 | *Panolis japonica* | Akechigahara,Shimojo, Nagano, Japan | 7. IV. 2011 | KJ194540 | KJ194551 | KJ194562 |
| H81 | *Panolis ningshan* **sp.nov.** | Qinling, Shaanxi, China | 05-V-2011 | KJ194537 | KJ194548 | KJ194559 |
| H300 | *Panolis estheri* | Qinling, Shaanxi, China | 18-IV-2012 | KJ194544 | KJ194555 | KJ194566 |
| H103 | *Panolis pinicortex* | Taoyuan, Taiwan, China | 12-III-2010 | KJ194541 | KJ194552 | KJ194563 |
| H104 | *Panolis exquisita* | Nanling, Guangdong, China | 08-IV-2011 | KJ194542 | KJ194553 | KJ194564 |
| H343 | *Panolis variegatoides* | Hualian, Taiwan, China | 17-III-2012 | KJ194543 | KJ194554 | KJ194565 |
| H272 | *Pseudopanolis heterogyna* | Qinling, Shaanxi, China | 18-IV-2012 | KJ194545 | KJ194556 | KJ194567 |
| H273 | *Pseudopanolis heterogyna* | Qinling, Shaanxi, China | 18-IV-2012 | KJ194546 | KJ194557 | KJ194568 |
| H278 | *Egira acronyctoides* | Taoyuan, Taiwan, China | 25-II-2010 | KJ194547 | KJ194558 | KJ194569 |
