# Supplementary material for: Phylogeny, Systematics and Biogeography of the Genus Panolis (Lepidoptera: Noctuidae) Based on Morphological and Molecular Evidence
Source: PLoS One. 2014 Mar 6;9(3):e90598. doi: 10.1371/journal.pone.0090598 (PMC3946178; doi:10.1371/journal.pone.0090598)
Supplement: Table S2 — Character matrix used in the morphological phylogenetic analysis. (DOCX) [file pone.0090598.s003.docx]

Table S2. Character matrix used in the morphological phylogenetic analysis

| Taxa/characters | 1 | 2 | 3 | 4 | 5 | 6 | 7 | 8 | 9 | 10 | 11 | 12 | 13 | 14 | 15 | 16 | 17 | 18 |
| --- | --- | --- | --- | --- | --- | --- | --- | --- | --- | --- | --- | --- | --- | --- | --- | --- | --- | --- |
| *E. acronyctoides* | 0 | 0 | 0 | 0 | 0 | 0 | 0 | 0 | 0 | 0 | 0 | 0 | 0 | 0 | 0 | 0 | 0 | 0 |
| *P. heterogyna* | 1 | 1 | 1 | 0 | 0 | 0 | 0 | 2 | 1 | 0 | 0 | 0 | 0 | 0 | 0 | 0 | 0 | 0 |
| *P. flammea* | 0 | 1 | 0 | 0 | 1 | 0 | 1 | 1 | 3 | 1 | 1 | 1 | 0 | 0 | 0 | 0 | 1 | 1 |
| *P. japonica* | 0 | 1 | 0 | 0 | 1 | 0 | 1 | 1 | 3 | 1 | 1 | 1 | 0 | 0 | 0 | 0 | 1 | 1 |
| *P.ningshan* | 0 | 1 | 0 | 0 | 0 | 1 | 1 | 1 | 3 | 0 | 1 | 1 | 1 | 0 | 0 | 0 | 0 | 1 |
| *P. estheri* | 0 | 1 | 0 | 0 | 0 | 0 | 1 | 1 | 3 | 0 | 1 | 1 | 0 | 0 | 0 | 1 | 0 | 1 |
| *P. pinicortex* | 0 | 1 | 1 | 0 | 0 | 2 | 1 | 2 | 2 | 0 | 1 | 0 | 0 | 0 | 1 | 0 | 0 | 1 |
| *P. exquisita* | 0 | 1 | 1 | 1 | 0 | 2 | 1 | 2 | 2 | 0 | 1 | 0 | 0 | 1 | 1 | 0 | 0 | 1 |
| *P. variegatoides* | 0 | 1 | 1 | 1 | 0 | 2 | 1 | 2 | 2 | 0 | 1 | 0 | 0 | 1 | 1 | 0 | 0 | 1 |
