# Supplementary material for: Phylogeny, Systematics and Biogeography of the Genus Panolis (Lepidoptera: Noctuidae) Based on Morphological and Molecular Evidence
Source: PLoS One. 2014 Mar 6;9(3):e90598. doi: 10.1371/journal.pone.0090598 (PMC3946178; doi:10.1371/journal.pone.0090598)
Supplement: Table S3 — Pairwise Kimura 2-Parameter (K2P) distances of COI sequences in all taxa used in this study. (DOCX) [file pone.0090598.s004.docx]

Table S3. Pairwise Kimura 2-Parameter (K2P) distances of *COI* sequences in all taxa used in this study

|  | 1 | 2 | 3 | 4 | 5 | 6 | 7 | 8 | 9 | 10 |
| --- | --- | --- | --- | --- | --- | --- | --- | --- | --- | --- |
| *Panolis flammea* H83 |  |  |  |  |  |  |  |  |  |  |
| *Panolis flammea* H100 | 0.00000 |  |  |  |  |  |  |  |  |  |
| *Panolis japonica* | 0.04567 | 0.04567 |  |  |  |  |  |  |  |  |
| *Panolis estheri* | 0.05381 | 0.05381 | 0.05068 |  |  |  |  |  |  |  |
| *Panolis ningshan* sp.nov. | 0.06363 | 0.06363 | 0.06711 | 0.06220 |  |  |  |  |  |  |
| *Panolis pinicortex* | 0.08027 | 0.08027 | 0.08217 | 0.07705 | 0.07700 |  |  |  |  |  |
| *Panolis exquisita* | 0.05864 | 0.05864 | 0.06040 | 0.05868 | 0.06208 | 0.04559 |  |  |  |  |
| *Panolis variegatoides* | 0.05868 | 0.05868 | 0.06723 | 0.05872 | 0.06553 | 0.04884 | 0.01855 |  |  |  |
| *Pseudopanolis heterogyna* H272 | 0.07362 | 0.07362 | 0.07201 | 0.07389 | 0.07410 | 0.08544 | 0.06865 | 0.07212 |  |  |
| *Pseudopanolis heterogyna* H273 | 0.07362 | 0.07362 | 0.07201 | 0.07389 | 0.07410 | 0.08544 | 0.06865 | 0.07212 | 0.00000 |  |
| *Egira acronyctoides* | 0.08915 | 0.08915 | 0.09283 | 0.09098 | 0.09445 | 0.09589 | 0.09292 | 0.08945 | 0.08578 | 0.08578 |
