# Supplementary material for: Phylogeny, Systematics and Biogeography of the Genus Panolis (Lepidoptera: Noctuidae) Based on Morphological and Molecular Evidence
Source: PLoS One. 2014 Mar 6;9(3):e90598. doi: 10.1371/journal.pone.0090598 (PMC3946178; doi:10.1371/journal.pone.0090598)
Supplement: Table S4 — The models and model parameters estimated with jModelTest. (DOCX) [file pone.0090598.s005.docx]

Table S4. The models and model parameters estimated with jModelTest

| Data  Name | Best-fit  model | Relative substitution rate | | | | | | Base frequencies | | | | PI  sites | Gamma shape |
| --- | --- | --- | --- | --- | --- | --- | --- | --- | --- | --- | --- | --- | --- |
|  |  | A-C | A-G | A-T | C-G | C-T | G-T | A | C | G | T |  |  |
| *COI* | TIM2+I | 6.7896 | 13.933 | 6.7896 | 1.0000 | 64.6167 | 1.0000 | 0.2897 | 0.1438 | 0.1525 | 0.4140 | 0.7570 | - |
| *16S* | TPM1uf+I+G | 1.0000 | 772.4928 | 307.6863 | 307.6863 | 772.4928 | 1.0000 | 0.3814 | 0.1438 | 0.0665 | 0.4084 | 0.3270 | 0.0760 |
| *EF-1a* | TrN+G | 1.0000 | 19.1219 | 1.0000 | 1.0000 | 7.0602 | 1.0000 | 0.2206 | 0.2428 | 0.2921 | 0.2445 | 0.0000 | 0.0140 |
| *COI*+*16S*+*EF-1a* | GTR+I+G | 2.7192 | 25.6469 | 15.1112 | 1.2682 | 46.7762 | 1.0000 | 0.2740 | 0.1966 | 0.2042 | 0.3253 | 0.5470 | 0.2850 |
